# Diagnostic Reference Levels based on clinical indications in computed tomography: a literature review

**DOI:** 10.1186/s13244-020-00899-y

**Published:** 2020-08-17

**Authors:** Graciano Paulo, John Damilakis, Virginia Tsapaki, Alexander A. Schegerer, Jacques Repussard, Werner Jaschke, Guy Frija, European Society of Radiology, Monika Hierath, Jonathan Clark

**Affiliations:** 1 ESTESC - Coimbra Health School, Medical Imaging and Radiotherapy Department, Instituto Politécnico de Coimbra, Rua 5 de Outubro, S. Martinho do Bispo, 3046-854 Coimbra, Portugal; 2 School of Medicine, University of Crete, Iraklion, Crete, Greece; 3 Konstantopoulio General Hospital, Athens, Greece; 4 Department of Medical and Occupational Radiation Protection, Federal Office for Radiation Protection, Neuherberg, Germany; 5 Radiation Protection and Image Processing Systems, Hirslanden AG, Glattpark, Switzerland; 6 International Consultant, St Nom la Bretèche, France; 7 Department of Radiology, Medical University Innsbruck, Innsbruck, Austria; 8 Paris Descartes University, Paris, France; 9 European Society of Radiology, Vienna, Austria

**Keywords:** Diagnostic Reference Levels, Computed tomography, Clinical indications, Computed tomography dose descriptors

## Abstract

**Background:**

In August 2017, the European Commission awarded the “European Study on Clinical Diagnostic Reference levels for X-ray Medical Imaging” project to the European Society of Radiology, to provide up-to-date Diagnostic Reference Levels based on clinical indications.

The aim of this work was to conduct an extensive literature review by analysing the most recent studies published and the data provided by the National Competent Authorities, to understand the current situation regarding Diagnostic Reference Levels based on clinical indications for computed tomography.

**Results:**

The literature review has identified 23 papers with Diagnostic Reference Levels based on clinical indications for computed tomography from 15 countries; 12 of them from Europe.

A total of 28 clinical indications for 6 anatomical areas (head, cervical spine/neck, chest, abdomen, abdomen-pelvis, chest-abdomen-pelvis) have been identified.

**Conclusions:**

In all the six anatomical areas for which Diagnostic Reference Levels based on clinical indications were found, a huge variation of computed tomography dose descriptor values was identified, providing evidence for a need to develop strategies to standardise and optimise computed tomography protocols.

## Key points

- The establishment, regular review and use of Diagnostic Reference Levels are mandatory according to the Council Directive 2013/59/EURATOM.
- Most of the existing Diagnostic Reference Levels have been established based on anatomical locations, which has some limitations as one could have several clinical indications with consequently different protocols corresponding to different exposure levels.
- In the anatomical areas for which Diagnostic Reference Levels based on clinical indications were found, a huge variation of computed tomography dose descriptors values has been identified.
- The EUCLID project aims to establish Diagnostic Reference Levels based on clinical indications.

## Background

The concept of Diagnostic Reference Levels (DRLs) was introduced many years ago by the International Commission on Radiological Protection (ICRP) [1] and has been widely accepted as a practical tool for optimisation in diagnostic and interventional radiology and nuclear medicine. DRLs should be used as a form of investigation level to identify unusually high dose levels. If DRLs are consistently exceeded, a local review usually takes place. DRLs are not intended for regulatory or commercial purposes, nor do they represent a dose constraint, nor are they linked to limits or constraints [2].

The European Union has formally introduced the concept and the mandatory use of DRLs in every Member State since 1997 [3], reinforcing the obligation for the establishment, regular review and use in 2013 through the Council Directive 2013/59/EURATOM (BSSD), on health protection of individuals against the dangers of ionising radiation in relation to medical exposure [4].

Most of the existing DRLs (independently of the imaging modality) have been established based on anatomical locations. However, some limitations of this approach were pointed out for computed tomography (CT) as, for the same anatomical location, one could have several clinical indications with consequently different protocols corresponding to different exposure levels. For example, chest CT could correspond to the work-up for pulmonary embolism, lung cancer, or even coronary calcium scoring, each of which requires corresponding image quality parameters and scan length, and hence should have different DRLs [5].

The clinical approach to DRLs was mentioned some years ago by the ICRP [6], but most of the European National Competent Authorities (NCAs) still consider DRLs for anatomical location and not for clinical indication. However, some countries have recently established DRL based on clinical indications (DRL_ci_) and some others are planning to do so in the near future. Also the European Society of Radiology (ESR) EuroSafe Imaging Call for Action 2018 has defined the objective to develop DRL_ci_ for adults and children, under action number 2 [7].

In this work, the dose descriptors used to define a DRL in CT are (a) volume computed tomography dose index (CTDI_vol_), the standard descriptor for estimating the output dose of a CT scanner, based on measurements obtained when scanning either a 16 cm or 32 cm phantom [8] and the unit used is mGy; (b) dose length product total (DLP_t_), which is the sum of the DLP values from each CT acquisition/phase, representing the measure of the total amount of radiation used to perform the CT examination. DLP is the product of the CTDI_vol_ (mGy) and scan length (cm), and the unit used is mGy.cm.

Both CTDI_vol_ and DLP_t_ are essential tools for CT optimisation; however it is important to understand the fact that they only represent CT scanner output and consequently are not patient dose estimates [9].

In August 2017, the European Commission (EC) launched the “European Study on Clinical Diagnostic Reference Levels for X-ray Medical Imaging” (EUCLID) project, to provide up-to-date DRL_CI_.

The main objectives of the EUCLID project, led by the ESR, were to conduct a European survey to collect data needed for the establishment of DRL_ci_ for the most important X-ray imaging tasks in Europe (from the radiation protection perspective) and to specify up-to-date DRL_ci_ for those examinations.

The aim of this work was to conduct an extensive literature review by analysing the most recent studies published and the data provided by NCAs, to understand the current situation regarding DRL_ci_ for CT, under the scope of EUCLID project.

## Materials and methods

One of the goals of EUCLID was the collection of information on the status of national DRLs and DRL_ci_ in Europe from NCAs from literature and from a workshop held in December 2019 in Luxembourg. The methodology for this included contacting the NCAs of 31 European countries and asking them to provide available national data that was then discussed and confirmed during the aforementioned workshop. Additionally, a comprehensive literature review was undertaken in order to identify which clinical indications had already been specifically studied.

To perform the literature review, several databases were used, such as science direct, PubMed and Google Scholar. Multiple keywords combination was used, such as diagnostic reference levels in computed tomography, clinical diagnostic reference levels and diagnostic reference levels based on clinical indications. All publications were collected and stored in the Mendeley reference management software (www.mendeley.com).

## Results

### Literature review for DRL_ci_ in CT

By using the keywords defined, data from 65 papers was considered and amongst them 23 included DRL_ci_, being that 3 of them were from countries outside Europe (United States of America, Japan and Egypt) and 12 from European countries: Austria, Denmark, Finland, France, Germany, Ireland, Italy, Norway, Sweden, Switzerland, The Netherlands, United Kingdom. In addition, data provided by the NCAs, discussed and validated during the workshop, were also included.

Considering that the concept of DRL_ci_ is a recent one, some discrepancy and inconsistency was found in the classification of the clinical indication.

The DRLci values found were for several anatomical areas and are listed in Table 1. A total of 28 clinical indications for 6 anatomical areas have been identified. The anatomical areas with the most values for DRL_ci_ were “head” and “abdomen”, with a total of 6 each.

**Table 1:** CT clinical indications

Anatomical area	Clinical indication
Head: *n*_ci_ 6	Acute stroke
	Haemorrhage/aneurysms/arteriovenous malformations
	Metastases/cerebral abscess
	Trauma
	Cholesteatoma
	Sinusitis
Cervical (spine and neck): *n*_ci_ 3	Fracture
	Disk pathology
	Adenopathy/abscesses
Chest: *n*_ci_ 5	Lung cancer
	Interstitial lung disease
	Pulmonary embolism
	Coronaries (CTC angiography)
	Coronaries (calcium scoring)
Abdomen: *n*_ci_ 6	Liver metastases
	Abscess
	Kidney stones/colic
	Kidney tumour/colic
	Acute abdomen
	Pancreas adenocarcinoma
Abdomen-pelvis: *n*_ci_ 5	Abscess/lymphadenopathy
	Virtual colonoscopy (polyps/tumour)
	Abdominal aorta angiography
	Colic
	Occlusion
Chest-abdomen-pelvis: *n*_ci_ 3	Tumour
	Infectious
	Oncologic follow-up

### Head CT

For head CT, 10 references with DRL_ci_ were found for 6 clinical indications: acute stroke; haemorrhage/aneurysms/arteriovenous malformations; metastases/cerebral abscess; trauma; cholesteatoma; sinusitis. Table 2 shows the DRL_ci_ for head CT with the CTDI_vol_ and/or DLP values for each clinical indication. The DRL_ci_ for trauma/sinusitis was the clinical indication with the most references found (7 out of 10). The DLP values ranged from 90 mGy.cm [14] to 1000 mGy.cm [18]. One publication presents DRL_ci_ for head CT both for males and females [16], demonstrating however similar or in some cases equal values.

**Table 2:** DRL_ci_ for head CT

Head CT																		
References	Acute stroke/post fossa		Acute stroke/cerebrum		Acute stroke/brain (whole)		Acute stroke/all sequences		Haemorrhage, aneurysms, arteriovenous malformations		Metastases, cerebral abscess		Trauma, sinusitis		Cholesteatoma		Sinusitis	
	CTDI_vol_ (mGy)	DLP (mGy.cm)	CTDI_vol_ (mGy)	DLP (mGy.cm)	CTDI_vol_ (mGy)	DLP (mGy.cm)	CTDI_vol_ (mGy)	DLP (mGy.cm)	CTDI_vol_ (mGy)	DLP (mGy.cm)	CTDI_vol_ (mGy)	DLP (mGy.cm)	CTDI_vol_ (mGy)	DLP (mGy.cm)	CTDI_vol_ (mGy)	DLP (mGy.cm)	CTDI_vol_ (mGy)	DLP (mGy.cm)
Danish Health Authority (DK) 2015 [10]	–	–	–	–	–	–	–	–	58	930	–	–	–	–	–	–	–	–
Public Health England (UK) 2016 [11]	80	–	60	–	60	–	–	970	–	–	–	–	–	–	–	–	–	–
Schegerer et al. (DE) 2017 [12]	–	–	–	–	–	–	–	–	–	–	–	–	9	120	–	–	–	–
Treier et al. (CH) 2010 (22)	–	–	–	–	–	–	–	–	65	1000	65	1000	25	350	50	250	–	–
Van der Molen et al. (NL) 2013 [13]	–	–	–	–	–	–	–	–	–	936	–	–	–	133	–	–	–	–
Wachabauer et al. (AT) 2017 [14]	–	–	–	–	–	–	–	–	–	–	–	–	–	90	–	–	–	–
Geryes et al. (FR) 2019 [15]	–	–	–	–	–	–	–	–	44	1010	44	790	43	920	–	–	–	–
Ireland (IE) MERU 2017 [16]	26 (a)	469 (a)	–	–	–	–	–	–	–	–	–	–	62 (a)	918 (a)	–	–	21 (a)	183 (a)
	31 (b)	477 (b)	–	–	–	–	–	–	–	–	–	–	64 (b)	927 (b)	–	–	21 (b)	210 (b)
Norway (NO) 2018 [17]	–	–	–	–	–	–	60	950	60	950	–	–	–	–	–	–	–	–
Sweden (SE) 2019 [18]	–	–	–	–	–	–	60	1000	60	1000	–	–	60	1000	–	–	–	-

^a^For female patients

^b^For male patients

### Cervical (spine and neck) CT

For cervical (spine and neck) CT, eight references with DRL_ci_ were found for three clinical indications: fracture, disk pathology and adenopathy/abscess. Table 3 shows the DRL_ci_ for cervical (spine and neck) CT with the CTDI_vol_ and/or DLP values for each clinical indication. The DRL_ci_ for fracture was the clinical indication with more references found (six out of eight). The DLP values ranged from 300 mGy.cm [18] to 640 mGy.cm [15]. One publication presents DRL_ci_ for head CT both for males and females [16], however, with similar values. Two publications from the same country, one from 2016 [11] and other from 2018 [19], show a reduction of DLP values from 600 mGy.cm to 440 mGy.cm for the same clinical indication “fracture”.

**Table 3:** DRL_ci_ for cervical CT

Cervical CT						
References	Fracture		Disk pathology		Adenopathy, abscesses	
	CTDI_vol_ (mGy)	DLP (mGy.cm)	CTDI_vol_ (mGy)	DLP (mGy.cm)	CTDI_vol_ (mGy)	DLP (mGy.cm)
Schegerer et al. (DE) 2019 [20]	20	–	25	–	–	–
Public Health England (UK) 2016 [11]	26	600	–	–	–	–
Treier et al. (CH) 2010 [21]	–	–	–	–	30	600
Geryes et al. (FR) 2019 [15]	31	640	–	–	–	–
Ireland (IE) MERU 2017 [16]	26 (a)	469 (a)	–	–	–	–
	31 (b)	477 (b)	–	–	–	–
Norway (NO) 2018 [17]	15	350	–	–	–	–
Sweden (SE) 2019 [18]	13	300	–	–	30	600
Public Health England (UK) 2018 [19]	21	440	–	–	–	–

^a^For female patients

^b^For male patients

### Chest CT

For chest CT, 23 references with DRL_ci_ were found for 6 clinical indications: lung cancer, interstitial lung disease, pulmonary embolism, coronary computed tomography angiography (CCTA), calcium scoring. Table 4 shows the DRL_ci_ for chest CT with the CTDI_vol_ and/or DLP values for each clinical indication. The DRL_ci_ for CCTA was the clinical indication with more references found (11 out of 23). The DLP values ranged from 170 mGy.cm [12] to 1400 mGy.cm [22]. One publication presents DRL_ci_ for CCTA [12] made with three different approaches: prospective, no padding, 170 mGy.cm (c); prospective, with padding, 280 mGy.cm (d); prospective, with gating, 380 mGy.cm (e). From the three approaches, the prospective, no padding technique is the one that provides the lowest DLP value (170 mGy.cm).

**Table 4:** DRL_ci_ for chest CT

Chest CT												
References	Lung cancer		Interstitial lung disease (axial)		Interstitial lung disease (helical)		Pulmonary embolism		CCTA		Calcium scoring	
	ctdi_vol_ (mgy)	dlp (mgy.cm)	ctdi_vol_ (mgy)	dlp (mgy.cm)	ctdi_vol_ (mgy)	dlp (mgy.cm)	ctdi_vol_ (mgy)	dlp (mgy.cm)	ctdi_vol_ (mgy)	dlp (mgy.cm)	ctdi_vol_ (mgy)	dlp (mgy.cm)
Castellano et al. (UK) 2017 [22]	–	–	–	–	–	–	–	–	–	173	–	–
Danish Health Authority (DK) 2015 [10]	16	620	–	–	13	500	–	–	29	230	–	–
Foley et al. (IE) 2012 [23]	–	–	7	276	–	–	13	432	–	–	–	–
Fukushima et al. (JP) 2012 [24]	–	–	–	–	–	–	–	–	–	1510	–	
Schegerer et al. (DE) 2019 [20]	–	–	–	–	–	–	–	–	20	330 (d)	–	
Hausleiter et al. 2009 [25]	–	–	–	–	–	–	–	–	69,6	1152	–	
Japan Network for Research on Medical Exposures (JP) 2015 [26]	–	–	–	–	–	–	–	–	90	1400	–	–
Kanal et al. (USA) 2017 [27]	–	–	–	–	–	–	19	557	–	–	–	–
Mafalanka et al. (FR) 2015 [28]	–	–	–	–	–	–	–	–	–	870	–	–
Palorini et al. (IT) 2014 [29]	–	–	–	–	–	–	–	–	–	1208	–	131
Public Health England (UK) 2016 [11]	12	610	4	140	12	350	13	440	–	–	–	–
Radiation and Nuclear Safety Authority (FI) 2013 [30]	11	430	–	–	–	–	–	–	–	–	–	–
Salama et al. (EG) 2017 [31]	–	–	–	–	22	421	–	–	–	–	–	–
Schegerer et al. (DE) 2017 [12]	–	–	–	–	–	–	15	300	36 (e) 19 (d)	551 (e) 270 (d)	8	119
Treier et al. (CH) 2010 [21]	–	–	–	–	–	–	–	–	–	1000	–	150
Van der Molen et al. (NL) 2013 [13]	–	–	–	–	–	276	–	371	–	671	–	51
Wachabauer et al. (AT) 2017 [14]	–	–	–	–	–	–	–	400	–	–	–	–
Geryes et al. (FR) 2019 [15]	–	–	–	–	–	–	8	310	–	–	–	–
Ireland (IE) MERU 2017 [16]	7 (a)	241 (a)	–	–	7 (a)	210 (a)	9 (a)	234 (a)	–	–	–	–
	7 (b)	272 (b)	–	–	7 (b)	249 (b)	12 (b)	278 (b)	–	–	–	–
Norway (NO) 2018 [17]	9	350	–	–	9	300	–	–	–	–	–	–
Sweden (SE) 2019 [18]	9	350	–	–	–	–	–		–	–	–	–
Public Health England (UK) 2018 [19]	–	–	–	–	–	–	–	–	–	170 (c)	–	–
	–	–	–	–	–	–	–	–	–	280 (d)	–	–
	–	–	–	–	–	–	–	–	–	380 (e)	–	–
Netherlands (NL) 2012 [32]	–	–	–	–	–	–	10	350	–	–	–	–

^a^For female patients

^b^For male patients

^c^Prospective, no padding

^d^Prospective, with padding

^e^Retrospective, with gating

### Abdominal CT

For abdominal CT, 11 references with DRL_ci_ were found for 6 clinical indications: liver metastasis, abscess, kidney stones/colic, kidney tumour/colic, acute abdomen and pancreas adenocarcinoma. Table 5 shows the DRL_ci_ for abdominal CT with the CTDI_vol_ and/or DLP values for each clinical indication. The DRL_ci_ for liver metastasis and kidney stone/colic were the clinical indications with more references found (6 out of 12). For liver metastasis, the DLP values ranged from 400 mGy.cm [15, 19] to 1423 mGy.cm [27]. For kidney stone/colic, the DLP values ranged from 200 mGy.cm [33] to 460 mGy.cm [18]. One publication presents DRL_ci_ for abdominal CT, both for males and females [16], demonstrating however similar values.

**Table 5:** DRL_ci_ for abdominal CT

Abdomen												
Reference	Liver metastases		Abscess		Kidney stones/colic		Kidney tumour/colic		Acute abdomen		Pancreas adeno Ca	
	CTDI_vol_ (mGy)	DLP (mGy.cm)	CTDI_vol_ (mGy)	DLP (mGy.cm)	CTDI_vol_ (mGy)	DLP (mGy.cm)	CTDI_vol_ (mGy)	DLP (mGy.cm)	CTDI_vol_ (mGy)	DLP (mGy.cm)	CTDI_vol_ (mGy)	DLP (mGy.cm)
Danish Health Authority (DK) 2015 [10]	–	–	–	–	–	–	–	–	17	700	–	–
Public Health England (UK) 2016 [11]	14	910	15	745	10	460	13	1150	–	–	–	–
Radiation and Nuclear Safety Authority (FI) 2013 [30]	–	–	–	–	7	330	–	–	–	–	–	–
Salama et al. (EG) 2017 [31]	31	1423	–	–	–	–	–	–	–	–	–	–
Treier et al. (CH) 2010 [21]	15	400	–	–	–	–	–	–	–	––	–	–
Van der Molen et al. (NL) 2013 [13]	–	–	–	–	–	329	–	1371	–	–	–	1000
Wachabauer et al. (AT) 2017 [14]	–	400	–	–	–	–	–	–	–	–	–	–
Ireland (IE) MERU 2017 [16]	9 (a)	554 (a)	–	–	6 (a)	254 (a)	–	–	–	–	–	–
	10 (b)	515 (b)	–	–	8 (b)	291 (b)	–	–	–	–	–	–
Norway (NO) 2018 [17]	–	–	–	–	5	250	13	1300	–	–	–	–
Sweden (SE) 2019 [18]	11	550	–	–	5	200	12	1000	–	–	–	–
Netherlands (NL) 2012 [32]	–	–	–	–	–	–	–	–	15	700	–	–

^a^For female patients

^b^For male patients

### Abdominopelvic CT

For abdominopelvic CT, five references with DRL_ci_ were found for five clinical indications: abscess/lymphadenopathy, virtual colonoscopy (VC)/polyps/tumour, CT for abdominal aortic aneurysms (AAA), colic and occlusion. Table 6 shows the DRL_ci_ for abdominopelvic CT with the CTDI_vol_ and/or DLP values for each clinical indication. The DRL_ci_ for abscess/lymphadenopathy was the clinical indication with more references found (four out of five). The DLP values ranged from 650 mGy.cm [11, 15, 34] to 750 mGy.cm [18].

**Table 6:** DRL_ci_ for abdomino-pelvis CT

Abdomino-pelvis CT										
References	Abscess lymphadenopathy		VC-polyps/tumour		CT angiography (AAA)		Colic		Occlusion	
	CTDI_vol_ (mGy)	DLP (mGy.cm)	CTDI_vol_ (mGy)	DLP (mGy.cm)	CTDI_vol_ (mGy)	DLP (mGy.cm)	CTDI_vol_ (mGy)	DLP (mGy.cm)	CTDI_vol_ (mGy)	DLP (mGy.cm)
Public Health England (UK) 2016 [11]	15	745	11	950	–	–	–	–	–	–
Treier et al. (CH) 2010 [21]	15	650	–	–	15	650	–	–	–	–
Van der Molen et al. (NL) 2013 [13]	–	–	–	–	–	727	–	–	–	–
Wachabauer et al. (AT) 2017 [14]	–	650	–	–	–	–	–	–	–	–
Geryes et al. (FR) 2019 [15]	–	650	–	–	–	–	8	400	12	880

### Chest-abdominopelvic CT

For chest-abdominopelvic CT, three references with DRL_ci_ were found for three clinical indications: tumour, infectious and oncologic follow-up. Table 7 shows the DRL_ci_ for chest-abdominopelvic CT with the CTDI_vol_ and/or DLP values for each clinical indication. The DRL_ci_ for tumour and oncologic follow-up were the clinical indications with more references found (two out of three). For tumour, the DLP values ranged from 870 mGy.cm [15] to 950 mGy.cm [35]. For oncologic follow-up, the DLP values ranged from 605 mGy.cm [36] to 970 mGy.cm [34]. One publication presents DRL_ci_ for abdominal CT, both for males and females [16], demonstrating however similar values.

**Table 7:** DRL_ci_ for chest-abdominopelvic CT

Chest abdomen pelvis						
References	Tumour		Infectious		Oncologic follow-up	
	CTDI_vol_ (mGy)	DLP (mGy.cm)	CTDI_vol_ (mGy)	DLP (mGy.cm)	CTDI_vol_ (mGy)	DLP (mGy.cm)
Geryes et al. (FR) 2019 [15]	10	870	11	970	11	970
Ireland (IE) MERU 2017 [16]	–	–	–	–	8 (a)	605 (a)
	–	–	–	–	8 (b)	643 (b)
Norway (NO) 2018 [17]	15	950	–	–	–	–

^a^For female patients

^b^For male patients

## Discussion

To our knowledge, this is the first article to perform literature review for DRL_ci_. Considering that DRL_ci_ is a recent concept, it is understandable that only a limited number of papers was found in the literature and most of the proposed DRL_ci_ came from the NCAs of 12 European countries.

In all the six anatomical areas where DRL_ci_ were found, a huge variation of CT dose descriptors values was identified, providing evidence that different approaches/protocols are used to perform the CT procedure for the same clinical indication.

In the 28 clinical indications identified in the literature, the procedures with the highest differences in DLP values were head trauma (11-fold), CCTA (9-fold), liver metastasis (3-fold) and cervical fracture (2-fold).

The huge variations in the reported CT dose descriptors values for almost all the clinical indications addressed above are likely to be explained by differences in protocols (exposure parameters and scan length), type and age of scanner, number of acquisition series and, in the specific case of CCTA, by the option of performing either prospective or retrospective acquisitions. The same variations in radiation doses for CT across patients is described in the literature, and the reasons are primarily related on how CT scanners are used [34], the differences in patient’s size (weight and height) [36] and to the level of image quality required to answer the clinical question [35]. Although the DRLs are defined for standard patients [4], taking into consideration that the weight and height of patients are also a determining factor for dose increase, categorising patients by body mass index should be considered in the near future [36].

Several other factors may also contribute to the heterogeneity of results shown in the DRL_ci_ tables. DLP values may refer to individual sequences or to a complete examination (total DLP), and in some cases, this information is not included in the paper/report.

In addition, different names have been used for what is likely to have been the same indication (e.g. abscess versus acute abdomen), and the question of whether these differences are related to various interpretations of the name of the clinical indication or to different practices remains open. A semantic refinement, with the precise description of the clinical indication, should be made in the future in order to minimise any variation related to the meaning of the clinical indication.

For liver metastases and a few other clinical indications, DRL_ci_ in terms of CTDI_vol_ are similar, but DRLs in terms of DLP differ considerably. The difference between results in values of total DLP (yet similar levels of CTDI_vol_) for examinations of the lower trunk could be a consequence of the present use of increased scan lengths and/or number of sequences (particularly in relation to imaging for different phases in the distribution of contrast medium). The substantial variations in CT protocols, for the same clinical indication, delivers several folds higher radiation than necessary [33].

Although a large number of research studies have shown that dose optimisation tools such as tube current modulation can reduce patient dose considerably, it is not known how these tools are being used in everyday clinical practice. Large differences in dose descriptors for the same clinical indication and, sometimes, for the same CT scanner model may be addressed by standardising acquisition protocols, using dose reduction tools properly and improving education of practitioners in medical radiation protection.

## Conclusions

From this literature review, it is obvious that there is a lot of space for improvement in terms of standardising the CT protocols for each clinical indication and that the development of European guidelines on this topic would be very useful as a tool to implement dose reduction strategies in CT procedures.

Continuing to develop DRLs for CT based in anatomical areas without taking into consideration the clinical indication will probably meet the minimum standard of the BSSD but will insufficiently contribute to fulfil the main purpose of the existence of DRLs: a tool for optimisation.

We expect that the results of this work can stimulate the radiological community and the NCAs to move toward the establishment of DRL_ci_ in a more harmonised and consistent way.

## Data Availability

Not applicable

## Abbreviations

BSSD: Council Directive 2013/59/EURATOM
CT: Computed tomography
CTDI_vol_: Volume computed tomography dose index
DLP_t_: Dose length product total
DRL_ci_: DRL based on clinical indications
DRLs: Diagnostic Reference Levels
EC: European Commission
ESR: European Society of Radiology
EUCLID: European Study on Clinical Diagnostic Reference Levels for X-ray medical Imaging
ICRP: International Commission on Radiological Protection
NCAs: National Competent Authorities
